# Differential Resting-State Connectivity Patterns of the Right Anterior and Posterior Dorsolateral Prefrontal Cortices (DLPFC) in Schizophrenia

**DOI:** 10.3389/fpsyt.2018.00211

**Published:** 2018-05-28

**Authors:** Natalia Chechko, Edna C. Cieslik, Veronika I. Müller, Thomas Nickl-Jockschat, Birgit Derntl, Lydia Kogler, André Aleman, Renaud Jardri, Iris E. Sommer, Oliver Gruber, Simon B. Eickhoff

**Affiliations:** ^1^Department of Psychiatry, Psychotherapy and Psychosomatics, RWTH Aachen University, Aachen, Germany; ^2^JARA BRAIN, RWTH Aachen University, Aachen, Germany; ^3^Institute of Systems Neuroscience, Medical Faculty, Heinrich Heine University Düsseldorf, Düsseldorf, Germany; ^4^Institute of Neuroscience and Medicine, Brain and Behaviour (INM-7), Research Centre Jülich, Jülich, Germany; ^5^Department of Psychiatry, Carver College of Medicine, University of Iowa, Iowa City, IA, United States; ^6^Iowa Neuroscience Institute, Carver College of Medicine, University of Iowa, Iowa City, IA, United States; ^7^Department of Psychiatry and Psychotherapy, Medical School, University of Tübingen, Tübingen, Germany; ^8^Werner Reichardt Center for Integrative Neuroscience, University of Tübingen, Tübingen, Germany; ^9^LEAD Graduate School and Research Network, University of Tübingen, Tübingen, Germany; ^10^Department of Neuroscience, University Medical Center Groningen, University of Groningen, Groningen, Netherlands; ^11^Univ Lille, CNRS UMR 9193, SCALab and CHU Lille, Division of Psychiatry, CURE platform, Fontan Hospital, Lille, France; ^12^Neuroscience Division, University Medical Centre Utrecht and Rudolf Magnus Institute for Neuroscience, Utrecht, Netherlands; ^13^Section for Experimental Psychopathology and Neuroimaging, Department of General Psychiatry, Heidelberg University, Heidelberg, Germany

**Keywords:** fMRI neuroimaging, Schizophrenia, DLPFC, resting-state fMRI, parcellation

## Abstract

In schizophrenia (SCZ), dysfunction of the dorsolateral prefrontal cortex (DLPFC) has been linked to the deficits in executive functions and attention. It has been suggested that, instead of considering the right DLPFC as a cohesive functional entity, it can be divided into two parts (anterior and posterior) based on its whole-brain connectivity patterns. Given these two subregions' differential association with cognitive processes, we investigated the functional connectivity (FC) profile of both subregions through resting-state data to determine whether they are differentially affected in SCZ. Resting-state magnetic resonance imaging (MRI) scans were obtained from 120 patients and 172 healthy controls (HC) at 6 different MRI sites. The results showed differential FC patterns for the anterior and posterior parts of the right executive control-related DLPFC in SCZ with the parietal, the temporal and the cerebellar regions, along with a convergent reduction of connectivity with the striatum and the occipital cortex. An increased psychopathology level was linked to a higher difference in posterior vs. anterior FC for the left IFG/anterior insula, regions involved in higher-order cognitive processes. In sum, the current analysis demonstrated that even between two neighboring clusters connectivity could be differentially disrupted in SCZ. Lacking the necessary anatomical specificity, such notions may in fact be detrimental to a proper understanding of SCZ pathophysiology.

## Introduction

In neuroimaging, the most widely used method of characterizing functional neuroanatomy is based on the integration of larger anatomical brain regions. Based on this methodology, the DLPFC is frequently treated as a unified region controlling function through a top-down modulation of task-relevant information processing in the premotor and posterior parietal associative cortices (1, 2).

The DLPFC is reciprocally interconnected with motor areas in the medial frontal lobe, the rostral cingulate cortex, the premotor cortices as well as the cerebellum and the superior colliculus (3–6). While there is consensus regarding the role of the DLPFC in executive control, the varied results with respect to the location and extent of activation sites [e.g., (7–9)] beg the question of whether this variability is due to the region's functional heterogeneity.

Deficits in executive function, working memory, and attention in schizophrenia [stable and common symptoms observed during the lifespan of schizophrenia patients (10–12) are thought to be linked to reduced activity in the bilateral] DLPFC and the dorsal parietal cortex (13). However, findings pertaining to DLPFC involvement in schizophrenia are not consistent with studies that do not reveal any differences in the DLPFC between HC (14) and others despite reporting patterns of hyperactivation (15). Moreover, there is considerable variability in terms of the location and extent of DLPFC impairment across functional neuroimaging experiments investigating executive functions in both healthy controls (16, 17) and patients (13, 18, 19). For instance, when healthy controls showed involvement of the BA44 and BA 40 in response to working memory challenge (17) the differences between patients with schizophrenia and healthy controls during working memory task were seen more posterior (BA 45, BA 46, BA 47) (18).

The inconclusive results of the studies mentioned above may be due not only to the variations in the experimental context but also to the lack of an understanding of the functional heterogeneity of the DLPFC region. This most likely indicates that the executive control of behavior relies on distinct DLPFC subregions involved in differentiable neural networks and cognitive functions.

**Figure 1:**
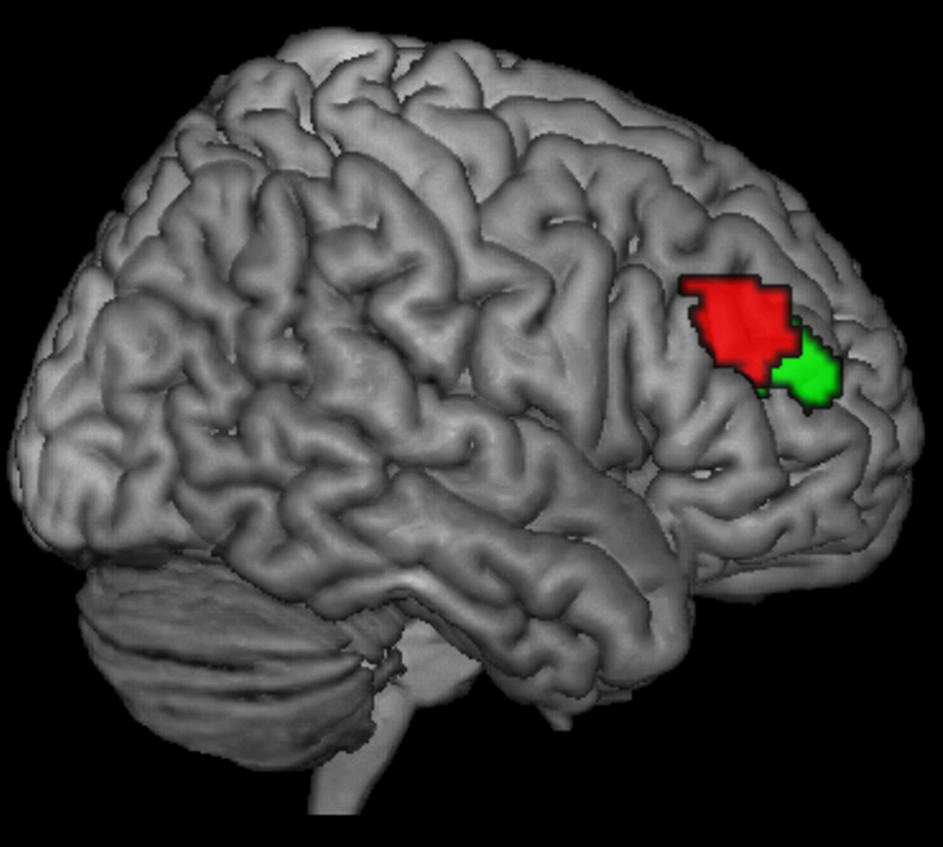
Posterior (red) and anterior (green) right DLPFC seed regions as derived from a previous co-activation-based parcellation study as described in Cieslik et al. (16).

The results of our previous parcellation study involving data from healthy individuals suggest that a single region of interest within the right DLPFC is not a functional entity but is organized hierarchically with one anterior ventral and one posterior dorsal DLPFC sub-region (Figure 1) (16). Our previous study showed an increased connectivity between the posterior (vs. anterior) DLPFC and the posterior parietal cortex, thereby indicating a possible network for cognitive control related to stimulus processing and selection of behavior-relevant information. In contrast, for the anterior (vs. posterior) DLPFC cluster, an increased functional connectivity with the ACC was seen. This resonates well with the results from a range of fMRI studies investigating cognitive control, suggesting that the DLPFC and the ACC (BAs 24 and 32) are specifically activated with an increase in demands for cognitive control and monitoring due to conflict in information processing and competing response plans (20–22). Thus, while the posterior cluster of the region is likely changed to be more strongly involved in action control processes, depending on the interaction with stimulus processing and working changed memory, the anterior region is likely responsible for higher-order control processes such as motor response monitoring and action inhibition (16).

Based on these observations, we sought to explore whether SCZ is linked to a differential connectivity disruption in the right anterior and posterior DLPFC (aDLPFC/pDLPFC) sub-regions, hypothesizing that a connectivity imbalance in the DLPFC sub-regions is part of the SCZ pathophysiology.

## Methods

### Definition of volume of interest (VOI)

The DLPFC VOIs were based on previous work (16), separating the right DLPFC region into two clusters by using hierarchical cluster analysis: a more anterior-ventral (center of gravity MNI coordinates: 30/43/23) one and a more posterior-dorsal one (center of gravity MNI coordinates: 37/33/32) (Figure 1). Henceforth, for the sake of simplicity, the regions will be referred to as anterior and posterior seeds or sub-regions. In the study by Cieslik et al. (16), volume of Interest was defined by merging the DLPFC activation sites from 4 previous studies investigating motor control. The first 3 studies (23, 24, 25) used manual stimulus–response tasks requiring a speeded response to a visual stimulus by a button press with either the left or right index finger, whereas the fourth study (26) used a manual sequence reproduction task. All 4 studies, which differed in the specific demands for executive motor control, showed activation in the right DLPFC with partially overlapping but slightly different locations. Following thresholding at *P* < 0.05 (cluster-level family-wise error [FWE]-corrected) of the individual contrasts, the ensuing 4 DLPFC clusters were combined into a single VOI (cluster size: 674 voxels). That is, every single voxel in the VOI region showed activation in at least one of the 4 studies. Subsequently, it was assessed whether this seed region could be divided into subregions based on similarities and differences between co-activation patterns of the individual seed voxels across neuroimaging experiments.

### Sample description

We investigated resting-state functional connectivity of the two right DLPFC sub-regions in 120 patients with a clinical diagnosis of schizophrenia [recruited at six sites: Aachen, Alberquerque (COBRE), Göttingen, Groningen, Lille, and Utrecht] and 172 healthy controls, with patients and controls not differing with respect to age Symptom severity was assessed by use of the Positive and Negative Syndrome Scale [PANSS, (27)] (for detailed demographic and clinical characteristics, please refer to Table 1 and Supporting Information Table S1).

**Table 1:** Demographic and clinical characteristics.

	**Controls (*n* = 172)**	**Patients (*n* = 120)**
Age (years)	33.55 ± 10.74	32.11 ± 9.37
Male/Female (*N*)	105/67	87/33
Duration of illness (years)		10.16 ± 9.51
POS (PANSS, positive)		15.08 ± 5.33
NEG (PANNS, negative)		14.68 ± 5.39
GEN (PANNS, general)		28.48 ± 8.07
PANSS (total)		58.32 ± 15.58

The diagnosis was ascertained by clinical examination of the attending psychiatrist according to the International Classification of Diseases (28) or the Diagnostic and Statistical Manual of Mental Disorders (29) criteria.

At the time of scanning, all patients were under antipsychotic medications, except for 6 (7%), who were not on any medication. Chlorpromazine-equivalents (CPZ-equivalents) were estimated as described in (Table 2) (30).

**Table 2:** Information on how many of the patients were medicated or unmedicated at time of measurement and mean medication (CPZ-equivalents) per site.

**Site**	**Unmedicated (*n*)**	**Medicated (*n*)**	Antidepressant^**^ (*n*)	**Mean CPZ-equivalents (SD)**
Site 1	0	9	2	572.22 (255.71)
Site 2	0	41	12	700.44 (368.29)
Site 3^*^	3	20	4	n.a^*^
Site 4	0	9	2	731.44 (617.83)
Site 5	1	9	5	820.44 (576.66)
Site 6	2	26	9	785.19 (493.64)
Total	6	114	34	795.63 (462.4)

*Site 1, Aachen; Site 2, COBRE; Site 3, Groningen; Site 4, Lille; Site 5, Utrecht; Site 6, Göttingen*.

* *Only statements regarding the nature of the drugs (irrespective of dose details) can be made here*.

** *All individuals taking antidepressants (combination of two or only one)*.

All subjects provided written informed consent to participate in the study prior to inclusion as approved by the ethics committees of the participating universities. Joint re-analysis was approved by the ethics committee at the Heinrich-Heine University Düsseldorf.

### Resting state fMRI data: imaging and preprocessing

For each subject resting state EPI (echo-planar-imaging) images were acquired using standard blood-oxygen-level-dependent (BOLD) contrast [gradient-echo EPI pulse sequence] (Please see Table S2 for details on EPI sequence for each site).

Prior to further processing (using SPM8, www.fil.ion.ucl.ac.uk/spm) the first four images were discarded allowing for magnetic field saturation. The EPI images were corrected for head movement by affine registration in a two-pass procedure realigning EPI volumes to its mean image. The mean EPI image for each subject was spatially normalized to the MNI ICBM-152 subject template using the “unified segmentation” approach (31). The ensuing deformation field was applied to the individual EPI volumes and smoothed with a 5-mm FWHM Gaussian kernel.

Neither the patient and control subsamples (sites) nor the overall disease cohorts showed group differences (*t*-tests with *p* > 0.2) in the three movement parameters (DVARS, FD, and RMD), indicating reasonably similar head motion during the scanning process (32).

To minimize spurious correlations between BOLD time courses through confounds such as physiological noise and motion, any variance that could be explained by the following nuisance variables was removed from each voxel's time series: (i) the six motion parameters derived from image realignment and (ii) their first derivative. According to published evaluations, motion regressors entered the model as first- and second-order terms resulting in 24 movement regressors (33) Given the evidence of group comparisons being distorted by correcting for the global mean signal (34, 35), the whole-brain resting-state functional connectivity of each of the two seeds (for all subjects) was calculated without Global Signal regression.

Finally, the data were band-pass filtered preserving BOLD frequencies between 0.01 and 0.08 Hz (36).

### Individual and group level analyses

For each subject and VOI the first eigenvariate of the VOIs time-series were calculated separately for each DLPFC seed and supplied to further whole-brain functional connectivity analysis. Linear (Pearson) correlation coefficients between the time series of the seed regions and those of all other gray-matter voxels in the brain were computed to quantify resting-state functional connectivity. These voxel-wise correlation coefficients were then transformed into Fisher's z-scores and then fed into a second-level analysis of variance (ANOVA).

Next, any variances from each voxel of the two seeds of the resting state functional connectivity (RSFC) data, which could be explained by age, gender, site and amount of within-scanner movement, were removed. To do so, voxel-wise effects of age, gender, site, and amount of within-scanner movement were included as predictors in a regression model based on only the HC data. This model was then used to adjust the FC data of each patient into relative scores. Therefore, these scores reflect hyper- or hypoconnectivity at each voxel relative to what would have been expected based on those covariates in HC. Thus, the deviations from the expected value calculated on the basis of HC values was used to express hyper- or hypoconnectivity of the DLPFC seeds in patients. The main problem that arises when estimating the coefficients for confounder adjustment from the entire group of subjects (i.e., patients and controls) is the potential multi-collinearity between socio-demographic confounders (age, gender as well as measurement site) and clinical characteristics and hence the neurobiological features of schizophrenia. Thus, the estimated effects of the confounders also include pathological features that are likely to introduce a bias to the estimation. In view of this, we resorted to the strategy described in Rozycki et al. (37), i.e., to estimate the effects of socio-demographic covariates only in the healthy control population and apply the ensuing model to adjust the data from all participants. Given that this approach permits an unbiased estimation of the effects of, e.g., age and gender, when it is applied to the patients, only the effects of the covariates (but not the disease process itself) ought to be removed. Similarly, the coefficients estimated from the model based on the HC data were applied to the whole sample including the patients. This effectively removed the influence of the demographic and, most importantly, any clinical effects on the difference between patients and controls. These adjusted data formed the basis of the between-group analysis (ANOVA) created to test for seed (aDLPFC/pDLPFC) × diagnostic group interaction.

### Resting state functional connectivity of the seed regions in HC

In a first step, we aimed to replicate the specific RSFC profile of the two DLPFC subregions that was found in our previous study (16). To do so, in the present healthy subsample we first tested for increased FC of the anterior vs. posterior seed (aDLPFC_HC > pDLPFC_HC) in conjunction with the main effect of the anterior seed's positive correlation (aDLPFC_HC). The same rational was done to test for the specific RSFC of the posterior seed (i.e., pDLPFC_HC > aDLPFC_HC ∩ pDLPFC_HC) in HC. The results were *p* < 0.05 family-wise error (FWE)-corrected on the voxel level.

### Group differences in whole-brain functional connectivity

In a conjunction analysis we tested for disorder-related changes in the connectivity of both seeds. To this end, we first identified regions to which both seed regions are connected in HC and for which both seeds show reduced connectivity in SCZ. To test this, a conjunction analysis across the difference in RSFC between HC and SCZ (HC > SCZ) for both seeds and the main effect of both seeds' positive correlation in HC (i.e., aDLPFC_HC > aDLPFC_SCZ ∩ pDLPFC_HC > pDLPFC_SCZ ∩ aDLPFC_HC ∩ pDLPFC_HC) was performed. Next, again using conjunction analysis, we looked for regions being significantly connected in HC and showing increased RSFC for SCZ compared to HC (i.e., aDLPFC_HC < aDLPFC_SCZ ∩ pDLPFC_HC < pDLPFC_SCZ ∩ aDLPFC_HC ∩ pDLPFC_HC). The results of both analyses, unless noted otherwise, were *p* < 0.05 family-wise error (FWE)-corrected on the voxel level.

Finally, we assessed seed × group interactions to reveal a specific disconnectivity of both DLPFC sub-regions. We tested for the regions showing a relatively increased positive RSFC with the posterior DLPFC cluster (and relatively decreased positive RSFC with the anterior cluster), and then visa-versa.

Results of the interaction were regarded as significant if they passed the threshold of a cluster-level FWE (cFWE) rate of *p* < 0.05 (cluster-forming threshold at voxel level: *p* < 0.001). The use of cFWE correction was on account of the fact that no significant clusters were left after FWE correction on the voxel level. The significant effects of the interaction were tested with *t*-tests. The results of the *t*-test were *p* < 0.05 family-wise error (FWE)-corrected on the voxel level.

### Correlations between functional connectivity and clinical parameters

At the next step we analyzed the relationship between the relative RSFC shifts between both seed regions and psychopathology in the schizophrenic sample. To this extend, the differences of adjusted RSFC between the anterior and posterior DLPFC seeds were calculated (pDLPFC > aDLPFC) in the SCZ sample. For this reason, positive differential values indicate a RSFC shift from the anterior (and toward the posterior) seed, while negative differential values indicate a RSFC shift from the posterior (and toward the anterior) seed. The calculated amount of differential disconnectivity was then correlated with the Positive and Negative Syndrome Scale (PANSS) total scores and all individual subscales. Again, the results were cFWE-corrected at *p* < 0.05 (Figures S1, S2, cluster-forming threshold at voxel level: *p* < 0.001). No significant clusters were left following additional Bonferroni correction. For exploratory reasons, results prior to Bonferroni correction have been provided.

### Anatomical allocation of results

Brain regions were anatomically allocated to probabilistic cytoarchitectonic maps using v2.0 of the SPM Anatomy Toolbox [http://www.fz-juelich.de/ime/spm_anatomy_toolbox; (38, 39)].

## Results

### Resting-state functional connectivity in HC

In HC, a significantly stronger RSFC of the posterior (compared to the anterior) seed was found with the left homotope (geometrically corresponding) region (peak MNI: −28/56/16; *T* = 9.48; 1,578 voxels), the bilateral IPS (peak MNI: 56, −34, 46/−44, −42, 42; areas hIP1, hIP2, and hIP3), the right inferior temporal gyrus (peak MNI: 60/–54/−16; *T* = 6.20; 123 voxels) and the left precentral gyrus (BA 44; peak MNI: −46/6/32; *T* = 6.90; 88 voxels). Conversely, significantly stronger RSFC of the anterior (compared to the posterior) seed was observed with the left homotope region (MNI-coordinates: −28/56/16 *T* = 9.48; 1,578 voxels), the left ACC (MNI-coordinates: −2/30/20; *T* = 7.18; 87 voxels), the left PCC (MNI-coordinates: −8/−46/28; *T* = 5.44; 66 voxels), the right inferior frontal gyrus (p. orbitalis, MNI-coordinates: 30/18/−22; *T* = 6.03; 64 voxels) and the right frontopolar region (Area Fp1, MNI-coordinates: 32/50/18; *T* = 40.58; 1,067 voxels).

Taken together, in a new sample of healthy controls we could replicate our previous results (16).

### Group differences in resting-state functional connectivity: conjunction analysis

**Figure 2:**
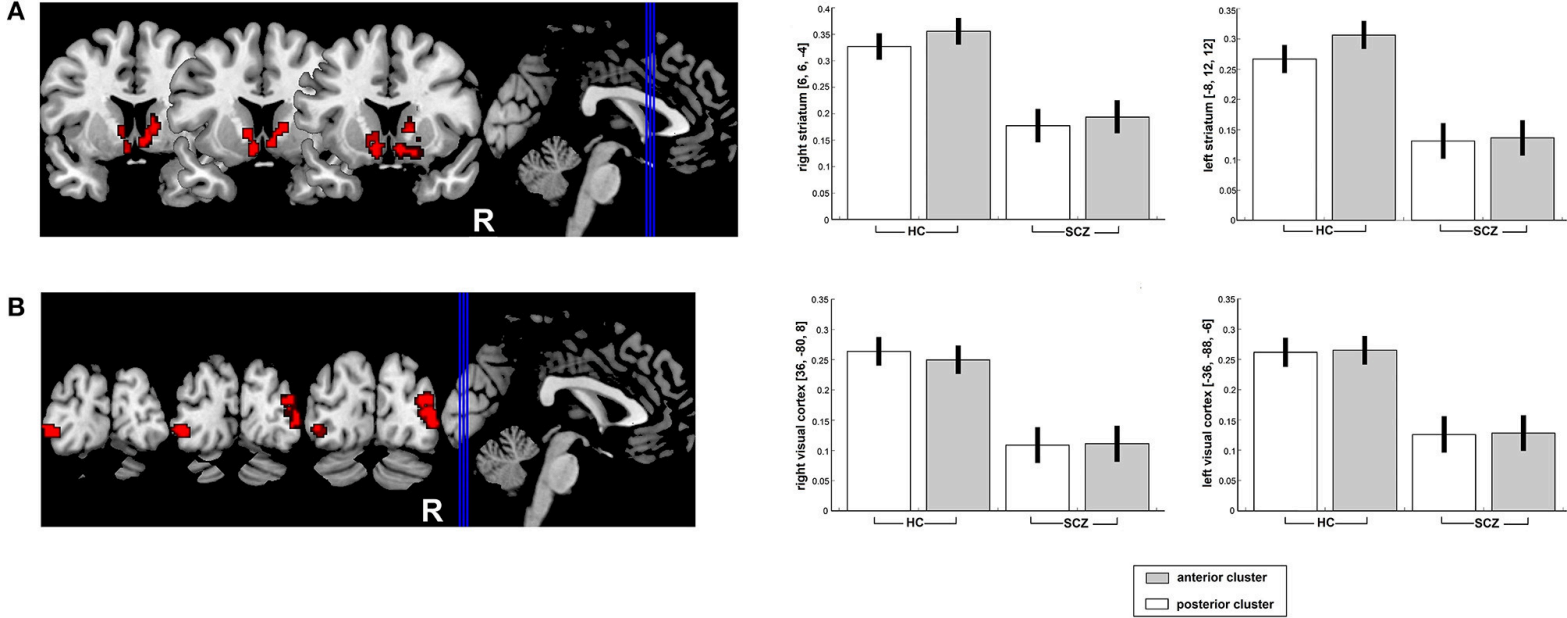
Significantly decreased functional connectivity of the posterior and anterior right DLPFC seeds (conjunction analysis) with the bilateral striatum **(A)** as well as the bilateral visual cortex **(B)** in SCZ patients as compared to HC. Results are projected onto the MNI single-subject template on the left side and contrast estimates for the effect in the point maximum respective region are shown on the right.

Compared to the HC, SCZ patients showed significantly reduced RSFC of the two seed regions (conjunction analysis) with the bilateral caudate, the right putamen, bilateral inferior occipital gyrus (V4) extending into middle occipital gyrus (Figure 2). For further details, please refer to Table 3.

**Table 3:** Regions with significantly decreased functional connectivity with both DLPFC seeds.

**Cluster**	**Voxel**	**Macro**	**Cyto**	***t*-score**	**MNI Coordinates**		
					**X**	**Y**	**Z**
1	317	R rectal gyrus		6.36	10	16	−12
		R caudate nucleus		6.23	6	6	−4
		R caudate nucleus		5.63	12	4	10
		R rectal gyrus		5.53	20	14	−14
		R rectal gyrus		5.52	18	18	−18
		R putamen		5.40	18	18	−6
2	198	R middle occipital gyrus extending to the inferior occipital gyrus	hOc4v [V4(v)]	6.15	36	−80	8
3	117	R putamen		5.93	32	−12	2
4	89	L caudate nucleus		5.95	−8	12	−12
5	67	L inferior occipital gyrus extending to the middle occipital gyrus	hOc4v [V4(v)]	5.96	−36	−88	−6
6	65	R inferior frontal gyrus		5.89	56	20	24
7	44	L cerebellum		5.29	−28	−90	−18
8	43	R inferior temporal gyrus		5.65	52	−54	−20
9	33	L middle occipital gyrus		5.36	−30	−90	8
10	13	R precuneus	7A	5.20	10	−68	62

Results of the individual contrasts [*p* < 0.05 family-wise error (FWE)-corrected on the voxel level] of SCZ-related reduction in RSFC with the anterior and posterior clusters respectively are presented in Tables S3, S4. Please also refer to Table S5 which shows for exploratory reasons the conjunction analysis results after cFWE correction. In contrast with these marked reductions, no significant RSFC increases were observed for either seed in SCZ patients relative to HC.

### Group differences in resting-state functional connectivity: group × seed interaction

**Figure 3:**
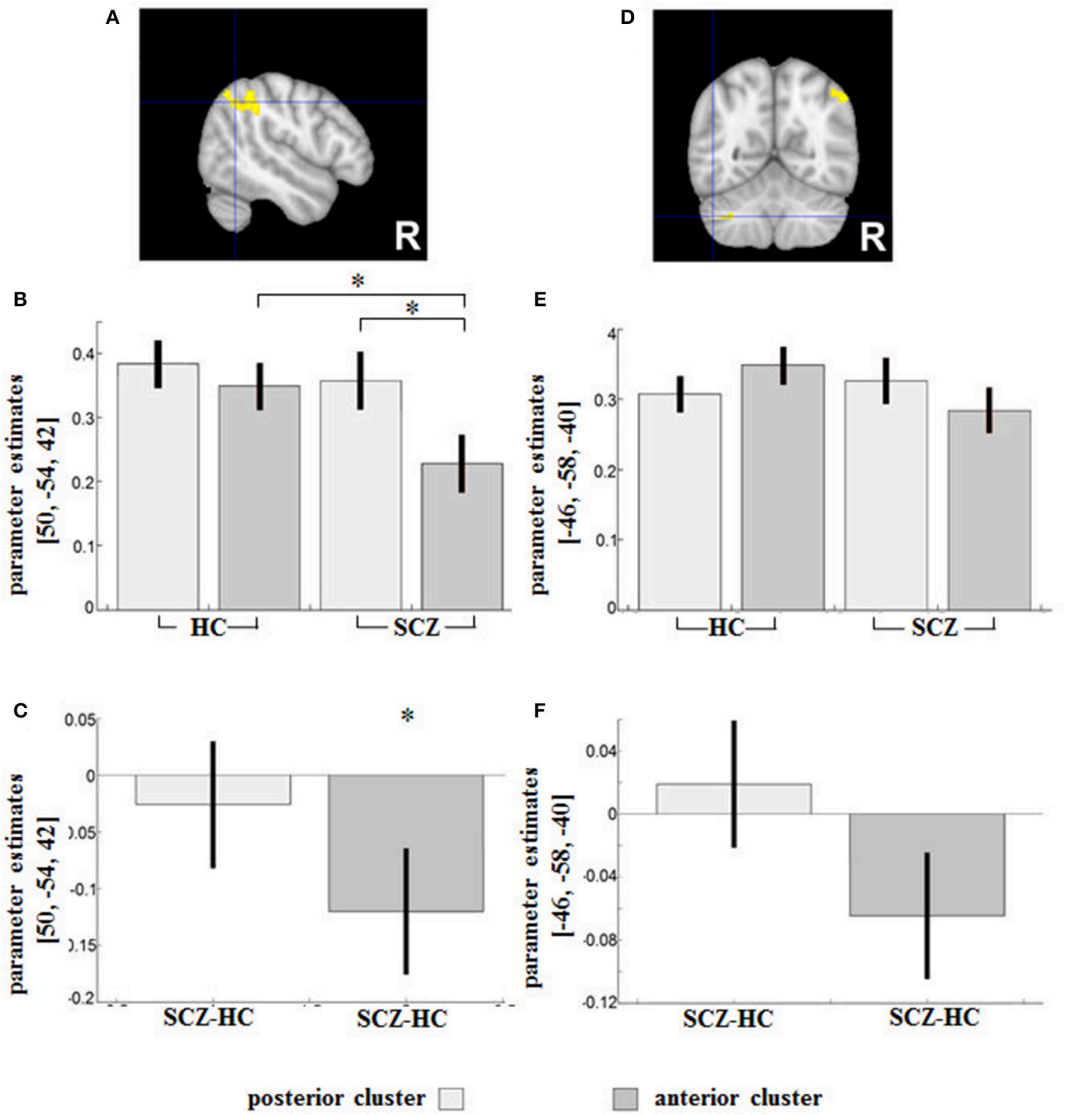
Regions showing an SCZ-related (relative) decreased functional connectivity with the anterior DLPFC compared to the posterior seed were **(A)** the right IPL (Area PGa) and **(D)** the left cerebellum. Contrast estimates for the effect in the respective region **(B,E)** and the difference (SCZ—HC) in RSFC between HC and SCZ for the posterior and anterior DLPFC seeds respectively **(C,F)** are demonstrated below. *indicates the significant effects of the interaction assessed with t-tests (*p* < 0.05 family-wise error (FWE)-corrected on the voxel level).

Seed (aDLPFC/pDLPFC) × diagnostic group (SCZ/HC) interactions indicated sub-regional DLPFC disconnectivity with the right inferior parietal lobule (IPL) (peak MNI: 50/−54/42; *T* = 3.74; 239 voxels) and the left cerebellum (peak MNI: −46/−58/−40; *T* = 4.62; 134 voxels) in patients (Figures 3A,D).

According to the parameter estimates (Figure 3B), the IPL of both groups had a weaker connectivity with the aDLPFC compared to the pDLPFC, although the difference was significant only in the patient group (peak MNI: 54/−34/60; *T* = 6.27) (Figure 3C).

In the second cluster located in the cerebellum, HC showed stronger FC in the anterior DLPFC compared to the posterior DLPFC, while a reverse pattern was observed in patients (Figure 3E). The group difference in parameter estimates (Figure 3F) indicated a shift of the cerebellum's RSFC away from the anterior (toward the posterior) DLPFC subregion among patients. None of the group comparisons revealed any significant differences in the region (Figure 3E).

**Figure 4:**
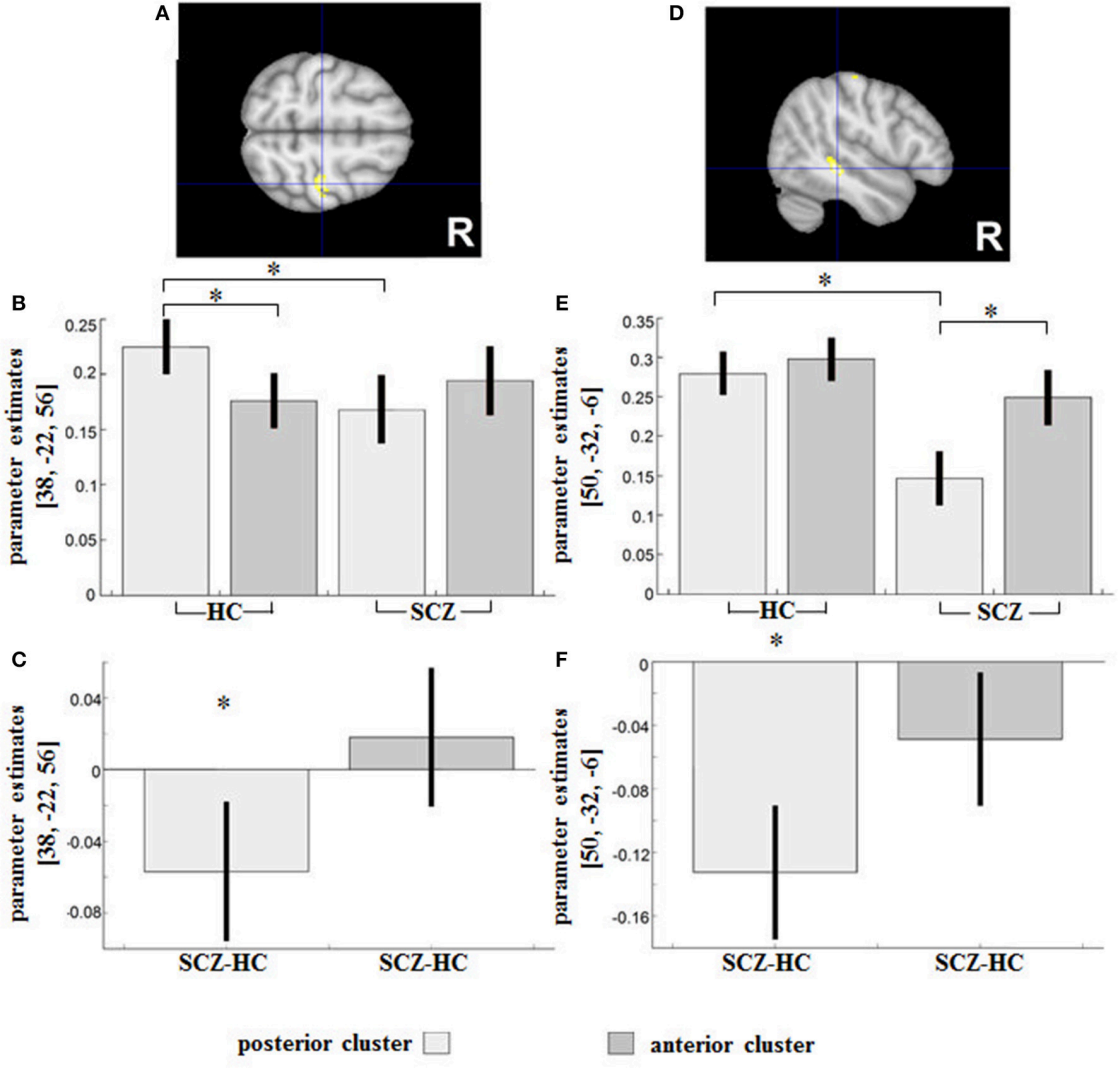
Regions showing an SCZ-related (relative) decreased functional connectivity with the posterior compared to the anterior DLPFC seed were **(A)** the right precentral gyrus and **(D)** the right middle temporal gyrus. Contrast estimates for the effect in the respective region are shown under **(B,E)**. Parameter estimates depict the difference in RSFC between HC and SCZ for the posterior and anterior DLPFC seeds respectively and are shown under **(C,F)**. *indicates the significant effects of the interaction assessed with t-tests (*p* < 0.05 family-wise error (FWE)-corrected on the voxel level).

The reverse interaction indicated further subregional DLPFC disconnectivity with the right precentral gyrus (Area 4a, peak MNI: 38/−22/56; *T* = 4.31; 127 voxels) and the right middle temporal gyrus (peak MNI: 50/−32/−6; *T* = 4.45; 115 voxels) (Figures 4A,D).

In HC, a significantly increased positive RSFC with the posterior DLPFC cluster (and decreased RSFC with the anterior cluster) was observed in Area 4a (peak MNI: 38/−22/56; *T* = 6.37), whereas a reverse non-significant relationship (relatively decreased positive RSFC with the posterior cluster and an increased RSFC with the anterior cluster) was observed in SCZ (Figure 4B).

The group difference in parameter estimates (Figure 4C) revealed an RSFC shift in patients from Area 4a toward the anterior and away from the posterior DLPFC subregion.

In the cluster located in the right middle temporal gyrus (Figure 4E), the SCZ group showed a relatively decreased positive RSFC with the posterior DLPFC cluster (and relatively increased positive RSFC with the anterior cluster), with these subregional differences in RSFC being statistically significant (peak MNI: 52/−30/−10; *T* = 7.23). The group difference in terms of parameter estimates (Figures 4E,F) revealed a significantly reduced positive RSFC between the right middle temporal gyrus and the posterior DLPFC (peak MNI: 48/−32/−6; *T* = 5.45; 20 voxels) in patients as compared to controls.

### Correlation with clinical parameters

With increasing symptom severity, several cortical regions showed a relatively decreased RSFC with the anterior DLPFC sub-region, paralleled by a relatively increased RSFC with the posterior sub-region.

**Figure 5:**
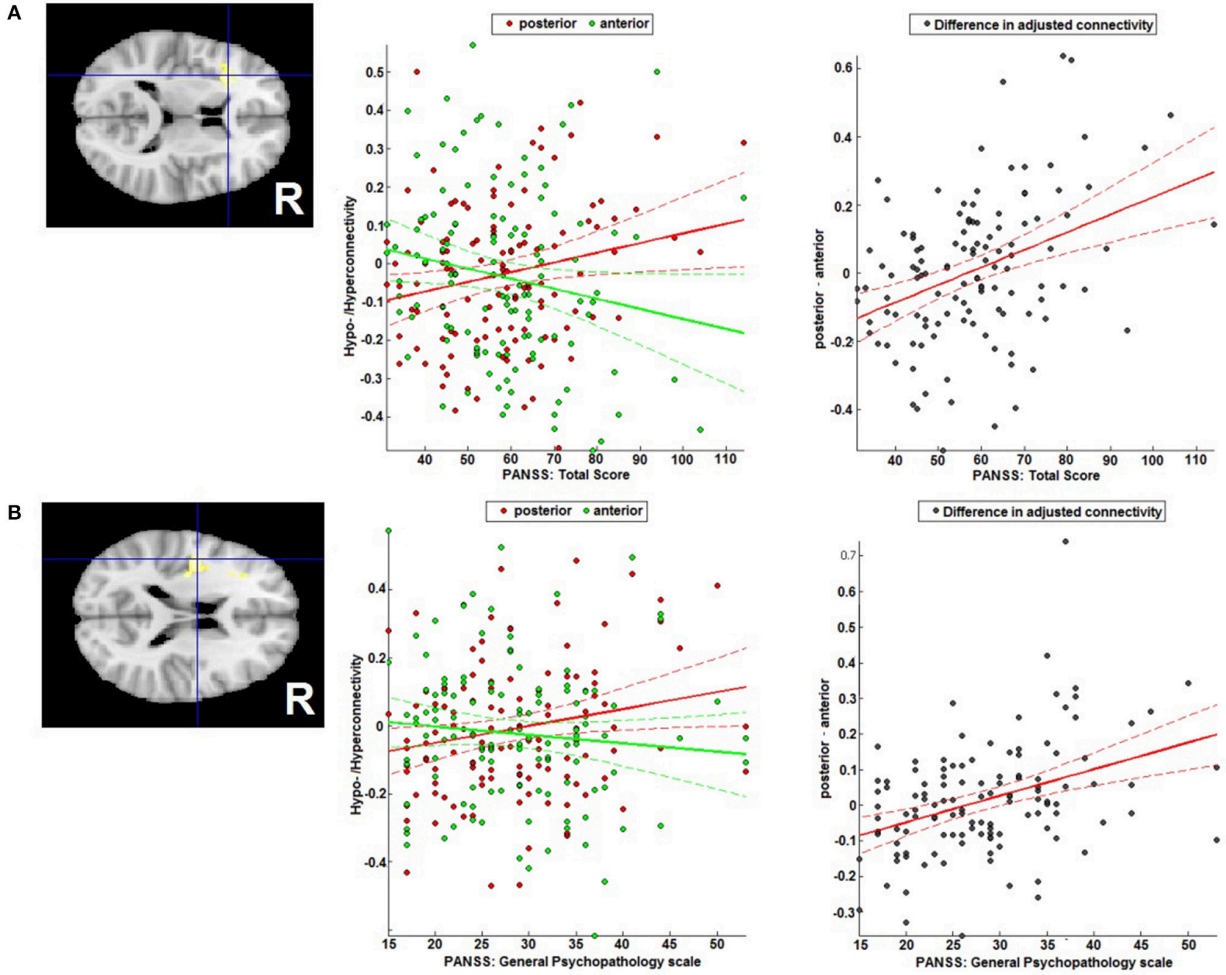
Correlation with symptomatology. Correlation of the difference between posterior and anterior DLPFC's FC with **(A)** the left IFG/anterior insula region and total PANSS score, and **(B)** the left operculum and the general PANSS score general PANSS score.

An increase in the difference in adjusted RSFC (calculated as pDLPFC > aDLPFC) in the left IFG/anterior insula lobe (MNI: −34/22/12; *T* = 4.35; 161 voxels) was found to go along with higher total PANSS scores (Figure 5A), while in the left operculum (MNI: −44/−6/18; *T* = 4.31; 124 voxels) a stronger difference was related to higher general psychopathology PANSS scores (Figure 5B).

The correlation analysis with the total negative symptoms scale and the age of onset did not reveal any significant effects.

## Discussion

We investigated RSFC in HC and patients with SCZ, targeting the connectivity for the right anterior and posterior executive control-related DLPFC sub-regions (16). In SCZ patients, those two neighboring sub-regions demonstrated diametrically altered functional connectivity affecting the right IPL, the left cerebellum, the right precentral gyrus and the right middle temporal gyrus. Correlation analysis indicated furthermore that the connectivity imbalance of the DLPFC sub-regions with the left IFG/anterior insula and the left operculum may be linked to symptom severity.

We believe that such small, albeit not well understood, effects have an enormous impact on the results (and their interpretation) of neuroimaging studies. The common practice of studying the DLPFC region as a whole obfuscates the differential effects, thus stymieing the progress of our understanding of DLPFC functions and their pathological effects in patient collectives.

### Reduction of RSFC with the striatum and the occipital cortex in both DLPFC sub-regions

Reduced corticostriatal connectivity, common among patients with chronic and adolescent-onset schizophrenia [(40, 41)] as well as patients with first-episode psychosis (42) and their relatives (43), is not only a trait characteristic, but also, as indicated by an improvement of psychosis symptoms with increased RSFC between the right striatum and the right DLPFC (44), a state characteristic of psychosis (44).

Reduced RSFC with the bilateral striatum in SCZ patients (compared to HC) was found for both DLPFC seeds. As corticostriatal dysconnectivity accounts for psychotic symptoms of SZC, it is not surprising that it likely affects rather large areas of the right DLPFC.

A reduced RSFC between the DLPFC and the bilateral V4 (a region crucial for visual object recognition and visual attention (45) is a less common finding in schizophrenia patients and is likely to be linked to disruptions in the visual attention network (46, 47).

### Attention and action inhibition: connectivity shifts from the anterior (toward the posterior) DLPFC subregion

Among patients, we observed a subregional DLPFC dysconnectivity with the right IPL and the left cerebellum. As previously shown by our group, the anterior DLPFC subregion in particular is likely involved in higher-order control processes of motor behavior such as attention and subsequent behavioral adjustments (16). As part of the frontoparietal executive-control network, the IPL is strongly interconnected with the DLPFC (48) and is also involved in attentional selection of sensory contents (49) and sustained attention (50). Our research (the presented work as well as the previous study by Cieslik et al. (16) has consistently shown an increased connectivity between the posterior (vs. anterior) DLPFC and the IPL in HC. In the present study, the IPL-aDLPFC connectivity was found to be weaker in patients compared to HC, and, only in patients, the IPL showed a significantly weaker connectivity to the aDLPFC compared to the pDLPFC. Apart from being linked to thought disorder and disruption of sensory integration, the reduced connection between the DLPFC (in particular the anterior DLPFC seed) and the IPL likely underlies the pronounced deficits in attention and other executive functions seen in psychosis (51). In addition, the connectivity shift from the anterior (toward the posterior) DLPFC region may underlie particular deficits in attentive stimulus processing and selection of behavior-relevant information. The connectivity shift may also explain why the involvement of the parietal cortex in SCZ strongly depends on the chosen task. For instance, compared to controls, patients with childhood-onset schizophrenia have shown significantly lower activation in the posterior parietal cortices along with decreased frontoparietal functional connectivity during a working-memory task (52). The link is underscored by the fact that selective attention plays a central role in working memory (53).

With cerebellar hypoactivation being commonplace in SCZ (54, 55), a few studies also indicate changes in the RSFC between the cerebellum and the DLPFC in SCZ (56, 57). The cerebellum, which has been known to be linked to the motor system, is also likely involved in attention, cognitive learning, and multisensory integration (58). It has also been seen to have a lower activation level during working-memory tasks in patients with childhood-onset schizophrenia (52). Habas et al. (59) have shown that the cerebellum, as part of cortico-cerebellar loops, is involved in executive control and salience detection. Our findings are also consistent with the notion of a misconnection in the cortico-cerebellar-thalamo-cortical (CCTC) network (60) underlying the pathophysiology of schizophrenia.

Taken together, our observations suggest that the cortico-cerebellar and cortico-parietal RSFC dysfunction in SCZ may not only be network-specific (61), but also strongly dependent on the exact localization of the DLPFC clusters. Given the involvement of the anterior DLPFC seed in higher-order control processes of motor behavior and attention (16), these findings can help broaden our understanding with respect to the task-based aberrant activity patterns in those regions.

### Action execution: connectivity shifts from the posterior (toward the anterior) DLPFC subregion

Here, we observed effects in the patients' primary motor cortex (Area 4a) and the right MTG.

The right DLPFC has been seen to be strongly interconnected with the motor cortex (4, 61, 62), modulating its activity during selection, planning, and execution of motor behavior (63). In HC, we saw a significantly increased positive RSFC with the posterior DLPFC cluster (and decreased RSFC with the anterior cluster) in Area 4a. This effect, likely associated in HC with a stronger emphasis of the posterior DLPFC cluster in action execution (16), was missing in patients. Connectivity shifts from the posterior (toward the anterior) DLPFC subregion and the Area 4a may be a correlate of reduced capacities in action execution (64) and prolonged motor planning and execution (65) seen in schizophrenia. The primary motor cortex, which is activated not only during execution (66, 67) but also during observation of others' actions (68), the latter putatively reflecting mirror neuron activity (69), is linked to a wide range of important social behaviors, from speech to imitation and empathy (69) and is reduced in schizophrenia (70, 71). The activation of the primary motor cortex is also closely linked to automatic motor potentiation: e.g., the viewing of a right-facing cup handle activates the left hemispheric motor areas (72), triggering potentiation of the right hand (73). The regulatory relationship between the frontal and primary motor cortices is also evident in what is known as “utilization behavior” (74), which is believed to be linked to intrusive, disruptive, and inappropriate motor behaviors seen in individuals with frontal system damage or dysfunction (e.g., dementias, schizophrenia, depressive disorders, attention deficit hyperactivity disorder) (75).

In the cluster located in the right MTG, only patients showed a connectivity shift from the posterior (toward the anterior) DLPFC. That this effect was not seen in HC, indicates its association with functional aberrations. Thus, the right MTG is linked to the perception of biological motion [movements generated by living beings; (76)] as well as different aspects of face perception (77, 78). In SCZ, deficits in face perception (79) and biological motion perception (80), processes crucial to the interpretation of social information, have already been elucidated (81). As demonstrated by our results, deficits in action execution in schizophrenia may be related in particular to the disturbed RSFC between the right DLPFC subregion and the primary motor cortices. Specifically, given the involvement of the posterior DLPFC seed in action execution (16), a connectivity shift from the posterior (toward the anterior) DLPFC may underlie impairments (in SCZ) with respect to prolonged motor planning and execution, or utilization behavior.

### The link between pathophysiology and psychopathology

The correlation analysis with psychopathology indicated that the sub-regional DLPFC disconnectivity with the left IFG/anterior insula and the left operculum correlated positively with increasing total PANSS and general psychopathology scores.

The ACC, the DLPFC, the insula and the posterior parietal cortex form a regulatory network for high-level cognitive control and attentional processes (82, 83). In particular, decreased gray matter in the anterior insula has been linked to executive function deficits observed in e.g., schizophrenia, bipolar disorder, and depression (84).

Patients with schizophrenia also experience difficulties in appropriately attributing self-generated sensory stimuli. The anterior insula/IFC region and the adjoining frontal operculum have been suggested to be involved in the processing of self-generated sensory stimuli (81). The regulatory influence of the right DLPFC on the anterior insula has been observed during the regulation of pain intensity (85) as well as visual and auditory awareness of the moment (81). Taken together, our findings suggest that symptoms linked to misperception of the self as a distinct entity, and also deficits in high-level cognitive control and attentional processes, are likely to be dependent on symptom severity.

## Limitations

Despite its careful design, and the fact that it reached its goal, the study had a few limitations that ought to be mentioned. First, the data were collected from 6 different sites, and thus the heterogeneity of scanners and sequences might potentially have affected the results. Second, given that the diagnosis was based (depending on the site where the subjects were recruited) on the DSM-5 or ICD-10 criteria, the procedure, despite the overlap in diagnostic criteria, could have led to diagnostic inconsistencies. Third, at the time of assessment, most patients (93%) were under psychopharmacological treatment, with about 69% (*n* = 83) of the patient's group taking multiple compounds and no more than six having the same combination of drugs. Therefore, the potential effect of medication on the results cannot be ruled out. Finally, another limitation of the study is the lack of neuropsychological assessment of patients, owing to which the examination of the DLPFC subregions in relation to executive functions was rendered impossible.

## Conclusion

As summarized, the current analysis involving a large, representative sample of SCZ patients has demonstrated that even the connectivity of two neighboring clusters identified by advanced *in-vivo* mapping may be differentially disrupted in SCZ. Thus, we conclude that the imbalanced connectivity of the sub-regions (anterior and posterior DLPFC seeds), rather than that of the DLPFC as a whole, characterizes the connectional disruption of the DLPFC in SCZ. We would also argue that prevalent concepts such as “prefrontal dysfunction” may be far too broad to help explain the apparently region-specific disturbances in SCZ.

## Author contributions

NC and SE: substantial contributions to the conception or design of the work; TN-J, BD, LK, AA, RJ, IS and OG: the acquisition of data for the work; NC, SE, EC, and VM: analysis and interpretation of data for the work; NC, SE, EC, and VM: drafting the work; NC, SE, EC, VM, TN-J, BD, LK, AA, RJ, IS, and OG: revising it critically for important intellectual content; NC, SE, EC, VM, TN-J, BD, LK, AA, RJ, IS, and OG: final approval of the version to be published; NC, SE, TN-J, BD, LK, AA, RJ, IS, and OG: agreement to be accountable for all aspects of the work in ensuring that questions related to the accuracy or integrity of any part of the work are appropriately investigated and resolved.

### Conflict of interest statement

The authors declare that the research was conducted in the absence of any commercial or financial relationships that could be construed as a potential conflict of interest.
