# Supplementary material for: Differential Resting-State Connectivity Patterns of the Right Anterior and Posterior Dorsolateral Prefrontal Cortices (DLPFC) in Schizophrenia
Source: Front Psychiatry. 2018 May 28;9:211. doi: 10.3389/fpsyt.2018.00211 (PMC5985714; doi:10.3389/fpsyt.2018.00211)
Supplement: Supplementary file 1 [file Table_1.DOCX]

Table S1

*Group Characteristics for Age and Gender*

| Site | | Age Mean (SD) | *p*-value | Males (*n*) | Females (*n*) | p-value |
| --- | --- | --- | --- | --- | --- | --- |
| Site 1 | Controls | 33.23 (11.97) | .775^a^ | 10 | 3 | 0.474 |
|  | Patients | 31.78 (11.02) |  | 8 | 1 |  |
| Site 2 | Controls | 34.98 (10.18) | .737^a^ | 42 | 22 | 0.053 |
|  | Patients | 34.27 (11.35) |  | 34 | 7 |  |
| Site 3 | Controls | 31.94 (11.21) | .414^a^ | 18 | 13 | 0.909 |
|  | Patients | 29.74 (7.16) |  | 13 | 10 |  |
| Site 4 | Controls | 29.07 (6.56) | .295^a^ | 5 | 10 | 1.000 |
|  | Patients | 31.67 (3.97) |  | 3 | 6 |  |
| Site 5 | Controls | 37.40 (13.99) | .229^a^ | 8 | 12 | 0.121 |
|  | Patients | 31.68 (6.08) |  | 7 | 3 |  |
| Site 6 | Controls | 31.93 (9.45) | .808^a^ | 22 | 7 | 0.807 |
|  | Patients | 31.32 (9.43) |  | 22 | 6 |  |
| All | Controls | 33.55 (10.74) | .237^a^ | 105 | 67 | 0.042 |
|  | Patients | 32.11 (9.37) |  | 87 | 33 |  |

*Note:* Site 1 = Aachen, Site 2 = COBRE, Site 3 = Groningen, Site 4 = Lille, Site 5 = Utrecht, Site 6 = Göttingen.
 ^a­^Statistical comparison performed via *t* test
