# Supplementary material for: Differential Resting-State Connectivity Patterns of the Right Anterior and Posterior Dorsolateral Prefrontal Cortices (DLPFC) in Schizophrenia
Source: Front Psychiatry. 2018 May 28;9:211. doi: 10.3389/fpsyt.2018.00211 (PMC5985714; doi:10.3389/fpsyt.2018.00211)
Supplement: Supplementary file 2 [file Table_2.DOCX]

Table S2

*Information on Scanning Sites Including Scanner Type and MRI Parameters*

| Site | Scanner | TR/TE (ms) | Number of Slices | Slice-Thickness (mm) | Gap (mm) | Flip Angle | Orientation | In-plane Resolution | Volumes Acquired |
| --- | --- | --- | --- | --- | --- | --- | --- | --- | --- |
| Site 1 | Siemens 3T TrioTim | 2000/28 | 34 | 3.3 | 0.3 | 77° | Axial | 3.6 x 3.6 mm² | 210 |
| Site 2 | Siemens 3T TrioTim | 2000/29 | 32 | 3.5 | - | 75° | Axial | 3.75 x 3.75mm² | 150 |
| Site 3 | Philips Interna Achieva 3T | 2400/28 | 43 | 3 | 0 | 85° | Axial | 3.44 x 3.44 mm² | 200 |
| Site 4 | IRM Philips 3T | 1000/9.6 | 45 | 3.4 | - | 9° | Axial | 3.2 x 3.2 mm² | 200 |
| Site 5ͣ* | Philips Achieva 3T | 21.75/32.4 | 40 | 4 | - | 10° | Coronal | 4 x 4 mm² | 600 |
| Site 6 | Siemens TrioTim | 2000/30 | 33 | 3 | 0.6 | 70° | Axial | 3 x 3 mm² | 156 |

*Note:* MRI, magnetic resonance imaging; TR, repetition time; TE, echo time. Site 1 = Aachen, Site 2 = COBRE, Site 3 = Groningen, Site 4 = Lille, Site 5 = Utrecht, Site 6 = Göttingen

*Utrect used a PRESTO-SENSE Sequence. This scan sequence achieves full brain coverage within 609 ms by combining a 3D-PRESTO pulse sequence with parallel imaging in 2 directions (8-channel SENSE headcoil).
