# Supplementary material for: Differential Resting-State Connectivity Patterns of the Right Anterior and Posterior Dorsolateral Prefrontal Cortices (DLPFC) in Schizophrenia
Source: Front Psychiatry. 2018 May 28;9:211. doi: 10.3389/fpsyt.2018.00211 (PMC5985714; doi:10.3389/fpsyt.2018.00211)
Supplement: Supplementary file 3 [file Table_3.DOCX]

Table S3

*Regions with significantly decreased functional connectivity with anterior right DLPFC seeds*

| Cluster | Voxel | Macro | Cyto | t-score | MNI Coordinates | | |
| --- | --- | --- | --- | --- | --- | --- | --- |
|  |  |  |  |  | X | Y | Z |
| 1 | 715 | R olfactory cortex  R olfactory cortex  R caudate nucleus  R rectal gyrus  R caudate nucleus |  | 7.37  7.20  7.05  6.87  6.58 | 8  14  8  18  14 | 16  16  8  16  12 | -12  -18  -2  -16  6 |
| 2 | 301 | L olfactory cortex  L superior orbital gyrus  L IFG (p. orbitalis)  L caudate nucleus |  | 7.61  7.08  5.76  5.55 | -8  -14  -22  -8 | 12  14  28  10 | -14  -18  -20  0 |
| 3 | 258 | R middle occipital gyrus  R middle occipital gyrus  R inferior occipital gyrus | hOc4v [V4(v)] | 6.23  6.15  5.36 | 40  36  36 | -84  -80  -78 | 0  8  -2 |
| 4 | 217 | R putamen  R putamen  R pallidum |  | 5.93  5.69  5.53 | 32  32  28 | -12  -18  -8 | 2  -2  0 |
| 5 | 145 | R IFG (p. triangularis)  R IFG (p. opercularis)  R IFG (p. triangularis) | Area 45  Area 45  Area 45 | 5.89  5.63  5.62 | 56  58  46 | 20  18  22 | 24  20  24 |
| 6 | 116 | L putamen  L pallidum  L pallidum  L thalamus | Prefrontal  Prefrontal | 5.46  5.39  5.38  5.09 | -30  -18  -24  -10 | -16  -6  -8  -14 | 2  0  0  2 |
| 7 | 108 | R middle orbital gyrus  R middle orbital gyrus  R middle orbital gyrus | Fp2  Fp2 | 5.80  5.77  5.72 | 10  12  16 | 56  58  46 | -6  -2  -4 |
| 8 | 81 | L Inferior Occipital Gyrus | hOc4v [V4(v)] | 6.03 | -36 | -88 | -6 |
| 9 | 73 | R cerebellum | Lobule VIIa crusI (Hem)85 | 6.15 | 14 | -88 | -30 |
| 10 | 71 | R IFG (p. Orbitalis) |  | 6.25 | 26 | 28 | -16 |
| 11 | 66 | R Inferior Temporal Gyrus |  | 6.22 | 54 | -54 | -22 |
