# Supplementary material for: Differential Resting-State Connectivity Patterns of the Right Anterior and Posterior Dorsolateral Prefrontal Cortices (DLPFC) in Schizophrenia
Source: Front Psychiatry. 2018 May 28;9:211. doi: 10.3389/fpsyt.2018.00211 (PMC5985714; doi:10.3389/fpsyt.2018.00211)
Supplement: Supplementary file 4 [file Table_4.DOCX]

Table S4

Regions with significantly decreased functional connectivity in patients with posterior right DLPFC seed

| Cluster | Voxel | Macro | Cyto | t-score | MNI Coordinates | | |
| --- | --- | --- | --- | --- | --- | --- | --- |
|  |  |  |  |  | X | Y | Z |
| 1 | 340 | R middle occipital gyrus  R middle occipital gyrus  R middle occipital gyrus | hOc4v [V4(v)] | 6.83  6.22  5.71 | 36  40  42 | -80  -84  -84 | 8  16  0 |
| 2 | 334 | R caudate nucleus  R caudate nucleus  R rectal gyrus  R putamen |  | 6.23  5.63  5.53  5.41 | 6  12  20  20 | 6  4  14  16 | -4  10  -14  -6 |
| 3 | 185 | L middle occipital gyrus  L middle occipital gyrus  L inferior occipital gyrus  L inferior occipital gyrus | hOc3v [V3v]  hOc4v [V4(v)]  hOc3v [V3v]  hOc4v [V4(v)] | 6.25  5.94  5.37  4.95 | -38  -42  -24  -28 | -88  -90  -98  -88 | -4  -2  -8  -10 |
| 4 | 185 | R putamen  R putamen |  | 6.61  5.87 | 34  32 | -12  -4 | 4  12 |
| 5 | 174 | L middle occipital gyrus  L middle occipital gyrus  L middle occipital gyrus | hOc4d [V3A]  hOc3v [V3v] | 6.12  6.02  5.15 | -28  -36  -30 | -88  -90  -80 | 10  8  16 |
| 6 | 124 | L inferior parietal lobule  L middle occipital gyrus | Area hIP3 (IPS) | 6.42  6.05 | -18  -24 | -62  -64 | 44  32 |
| 7 | 107 | L caudate nucleus  L caudate nucleus  L rectal gyrus  L caudate nucleus  L putamen |  | 5.95  5.43  5.30  5.23  5.00 | -8  -8  -14  -10  -14 | 12  10  18  8  12 | -12  0  -14  4  -6 |
| 8 | 101 | R Inferior Occipital Gyrus | hOc4v [V4(v)] | 5.68 | 46 | -76 | -16 |
| 9 | 96 | L Lingual Gyrus | hOc4v [V4(v)] | 5.98 | -24 | -88 | -18 |
| 10 | 84 | R Inferior Temporal Gyrus | Area FG2 | 5.94 | 52 | -62 | -20 |
| 11 | 82 | R IFG (p. Triangularis) | BA 45 | 6.26 | 56 | 20 | 22 |
| 12 | 82 | L Inferior Temporal Gyrus |  | 6.05 | -46 | -62 | -6 |
