# Supplementary material for: Differential Resting-State Connectivity Patterns of the Right Anterior and Posterior Dorsolateral Prefrontal Cortices (DLPFC) in Schizophrenia
Source: Front Psychiatry. 2018 May 28;9:211. doi: 10.3389/fpsyt.2018.00211 (PMC5985714; doi:10.3389/fpsyt.2018.00211)
Supplement: Supplementary file 5 [file Table_5.DOCX]

Table S5

Regions with significantly decreased functional connectivity with both DLPFC seeds (conjunction analysis) after cFWE correction

| Cluster | Voxel | Macro | Cyto | t-score | MNI Coordinates | | |
| --- | --- | --- | --- | --- | --- | --- | --- |
|  |  |  |  |  | X | Y | Z |
| 1 | 17562 | R Caudate Nucleus  R Middle Occipital Gyrus  L Inferior Occipital Gyrus  L Caudate Nucleus |  | 6.23  6.15  5.96  5.95 | 6  36  -36  -8 | 6  -80  -88  12 | -4  8  -6  -12 |
| 2 | 976 | R IFG (p. Triangularis) | Area 45 | 5.89 | 56 | 20 | 24 |
| 3 | 740 | R Precuneus | Area 7A (SPL) | 5.21 | 10 | -68 | 62 |
| 4 | 424 | L IFG (p. Opercularis)  L Precentral Gyrus | Area 44 | 4.85  4.53 | -56  -52 | 14  4 | 24  36 |
| 5 | 286 | R Medial Temporal Pole |  | 5.61 | 50 | -18 | -28 |
| 6 | 248 | R Precentral Gyrus |  | 4.75 | 38 | 0 | 50 |
| 7 | 236 | L IFG (p. Triangularis) | Area 45 | 4.24 | -54 | 36 | 2 |
| 8 | 218 | R Mid Orbital Gyrus  R ACC | Area Fp2 | 4.61  4.22 | 12  6 | 56  42 | -2  -2 |
| 9 | 213 | R Fusiform Gyrus  R Inferior Temporal Gyrus |  | 4.78  4.60 | 38  44 | -16  -14 | -28  -32 |
| 10 | 139 | L Calcarine Gyrus  L Linual Gyrus | hOC1 [V1] | 4.90  4.06 | -14  -4 | -54  -62 | 4  6 |
