# Supplementary material for: Differential Resting-State Connectivity Patterns of the Right Anterior and Posterior Dorsolateral Prefrontal Cortices (DLPFC) in Schizophrenia
Source: Front Psychiatry. 2018 May 28;9:211. doi: 10.3389/fpsyt.2018.00211 (PMC5985714; doi:10.3389/fpsyt.2018.00211)
Supplement: Supplementary file 6 [file Data_Sheet_1.DOCX]

**Supplemental**

Disease-related RSFC changes in each seed region were tested relative to HC in conjunction with the main effect of the respective seed's positive correlation in the HC group (i.e. i) aDLPFC_HC > aDLPFC_SCZ ∩ aDLPFC_HC for decreased FC of the anterior DLPFC seed region in SCZ compared to HC and ii) pDLPFC_HC > pDLPFC_SCZ ∩ pDLPFC_HC for decreased RSFC of the posterior DLPFC seed region in SCZ compared to HC). Results of this analysis were p<0.05 family-wise error (FWE)-corrected on the voxel level, with a cluster-extent threshold of 60 voxels. As indicated by conjunction analysis across both contrasts, effects located in the right nucleus caudatus, the right putamen, the right middle occipital gyrus and the right IFG (pars triangularis) were seen in both contrasts (please refer to the results section).

For the anterior DLPFC cluster, we additionally found a reduced RSFC (in SCZ compared to HC) with the bilateral olfactory cortex as well as the left putamen, the right middle orbital gyrus, the right cerebellum, the right IFG (pars orbitalis) and the right inferior temporal gyrus. For the posterior DLPFC cluster, on the other hand, SCZ additionally showed reduced RSFC compared to HC with the left middle occipital gyrus, the left superior parietal lobule, the left caudate nucleus, the right inferior occipital gyrus, the left lingual gyrus and the bilateral inferior temporal gyri.

**Correlation with clinical parameters**

The correlation analysis with several single items of the PANSS revealed that, with increasing symptom severity, several cortical regions had an increased RSFC with the posterior DLPFC sub-region as well as a decreased RSFC with the anterior DLPFC sub-region. Consequently, the resulted differences in the adjusted RSFC (calculated as pDLPFC > aDLPFC) positively correlated with increasing psychopathology scores. In the correlation analysis with the item “delusion”, this pattern of sub-regional DLPFC disconnectivity was seen in the Area 2 (peak MNI: 28/-44/46; T=4.46; 148 voxels) (Sub. Figure 1A); in the correlation analysis involving the item “conceptual disorganization” it was seen in the right postcentral gyrus (peak MNI: 16/-26/48; T=5.05; 314 voxels) and the left IFG (peak MNI: -44/26/-12; T=4.64; 249 voxels) (Sub. Figure 1BC). In the analysis with the item "difficulty in abstract thinking", it was seen in the left IFG (p. Opercularis, peak MNI: -38/14/20; T=5.07; 296 voxels) and the left middle frontal gyrus (peak MNI: -46/46/0; T=4.80; 127 voxels) (Sub. Figure 2AB) and, finally, in the correlation analysis with the item "lack of spontaneity and flow of conversation," in the left middle occipital gyrus (peak MNI: -32/-80/20; T=4.68; 356 voxels) and the left IFG (p. Triangularis, peak MNI: -44/44/2; T=4.35; 159 voxels) (Sub. Figure 2CD).

Sup. Figure 1 Correlation with positive symptoms

Regions showing significant correlation between respective clinical parameters (Delusion and Conceptual disorganization) and a shift away from the anterior DLPFC FC were the A) primary somatosensory cortex (Area 2), B) the postcentral gyrus (Area 4a) extending into the right MCC and C) the left inferior frontal gyrus. Results are projected onto the MNI single-subject template.

Sup. Figure 2 Correlation with negative symptoms

Regions showing significant correlation between respective clinical parameters (Difficulty in abstract thinking and Lack of spontaneity and flow of conversation) and a shift away from the anterior DLPFC were A) the left inferior frontal gyrus (p. Opercularis), B) the left middle frontal gyrus, C) the left middle occipital gyrus and D) the left inferior frontal gyrus (p. Triangularis). Results are projected onto the MNI single-subject template.
